# Supplementary material for: Canine Adipose-Derived Mesenchymal Stromal Cells Reduce Cell Viability and Migration of Metastatic Canine Oral Melanoma Cell Lines In Vitro
Source: Vet Sci. 2024 Dec 9;11(12):636. doi: 10.3390/vetsci11120636 (PMC11680336; doi:10.3390/vetsci11120636)
Supplement: Supplementary file 1 [file vetsci-11-00636-s001.zip › Table S1.pdf]

Supplementary data S1

Table S1. Clinical and epidemiological information from cell donor patients

Cell line	Age	Animal	Tissue origin	Condition
MeLn (High-passage)	12 years old	Dog (Yorkshire)	Metastatic lymph node	Canine oral melanoma
UNESP-MEL3 (Low-passage)	10 years old	Dog (Teckel)	Metastatic lymph node	Canine oral melanoma
Ad-MSC	-	Female dog	Inguinal subcutaneous fat	Health
PBMC	-	3 health dogs	Canine peripheral blood	Health
